# Fluid Flow along Venous Adventitia in Rabbits: Is It a Potential Drainage System Complementary to Vascular Circulations?

**DOI:** 10.1371/journal.pone.0041395

**Published:** 2012-07-26

**Authors:** Hong-yi Li, Min Chen, Jie-fu Yang, Chong-qing Yang, Liang Xu, Fang Wang, Jia-bin Tong, You Lv, Caidan Suonan

**Affiliations:** 1 Cardiology Division, Beijing Hospital of the Ministry of Health, Beijing, People’s Republic of China; 2 Radiology Division, Beijing Hospital of the Ministry of Health, Beijing, People’s Republic of China; 3 Pathology Division, Beijing Hospital of the Ministry of Health, Beijing, People’s Republic of China; 4 Dipping Therapy Department, Qinhai Provincial Tibetan Hospital, Xining, People’s Republic of China; University of Arizona, United States of America

## Abstract

**Background:**

Our previous research and other studies with radiotracers showed evidence of a centripetal drainage pathway, separate from blood or lymphatic vessels, that can be visualized when a small amount of low molecular weight tracer is injected subcutaneously into a given region on skin of humans. In order to further characterize this interesting biological phenomenon, animal experiments are designed to elucidate histological and physiologic characteristics of these visualized pathways.

**Methods:**

Multiple tracers are injected subcutaneously into an acupuncture point of KI3 to visualize centripetal pathways by magnetic resonance imaging or fluorescein photography in 85 healthy rabbits. The pathways are compared with venography and indirect lymphangiography. Fluid flow through the pathways is observed by methods of altering their hydrated state, hydrolyzing by different collagenases, and histology is elucidated by optical, fluorescein and electron microscopy.

**Results:**

Histological and magnetic imaging examinations of these visualized pathways show they consist of perivenous loose connective tissues. As evidenced by examinations of tracers’ uptake, they appear to function as a draining pathway for free interstitial fluid. Fluorescein sodium from KI3 is found in the pathways of hind limbs and segments of the small intestines, partial pulmonary veins and results in pericardial effusion, suggesting systematical involvement of this perivenous pathway. The hydraulic conductivity of these pathways can be compromised by the collapse of their fiber-rich beds hydrolyzed by either of collagenase type I, III, IV or V.

**Conclusions:**

The identification of pathways comprising perivenous loose connective tissues with a high hydraulic conductivity draining interstitial fluid in hind limbs of a mammal suggests a potential drainage system complementary to vascular circulations. These findings may provide new insights into a systematically distributed collagenous connective tissue with a circulatory function and their potential relevance to the nature of acupuncture meridians.

## Introduction

The search for the anatomical and histological structures of the ancient meridians has continued for decades. Trying to identify their scientific basis, previous studies have anatomically revealed neural pathways, connective tissue facial planes, collagen fiber bands [1] and Bonghan ducts (BHDs) [2]. Other studies have focused on functional correlates including reduced electrical impedance and enhanced migration of nuclear tracers. [3], [4] Given these two reported functions, the electric properties have been studied since the 1950s and anatomically linked with not only neurovascular bundles and nervous system, but also inter-muscular connective tissue planes and collagenous connective tissues. [3] By contrast, the other function of transporting tracers has never linked with a definitive anatomical structure until now.

In parallel with the neural hypothesis, since 1980s, several studies [5]–[7] have demonstrated an interesting biological phenomenon in which a linear migration pathway of interstitial fluid within loose connective tissue is visualized by injecting a low molecular weight (MW) radiotracer subcutaneously, usually at an acupuncture point. For areas where the tracer is injected at nearby controls, the migration is not observed. [7] According to various experiments, the interpretation of results is possibly due to “perivascular-like-spaces” histologically [8] and not directly attributable to lymphatics or blood vessels.[6]–[8] Recently in our previous studies [9], by injecting a small amount of low MW paramagnetic contrast, Gadopentetate Acid Dimeglumine (Gd-DTPA, MW is 938) (Magnevist, Schering China Limited) into six acupuncture points, we have also clearly visualized six subcutaneous pathways in humans compared to adjacent non-acupuncture control points which had a characteristic “puncture-resistant” appearance, indicating a non-tubular structure, not consistent with lymphatic or blood vessels.

According to known physiology, the injected low MW tracers would mix with subcutaneous interstitial fluid in an acupuncture point and should be drained centripetally into adjacent lymphatic or vascular tree. Thus, what kinds of anatomical structures have centripetally drained the interstitial fluid from an acupuncture point? Are those blood or lymphatic vessels?

In order to study the anatomic and histologic structure of the draining pathways of interstitial fluid in mammals, I have designed animal experiments using the low MW extracellular fluid tracers Gd-DTPA [10] and fluorescein sodium (MW is 376.28) (WuZhou Zhongheng Group Co., Ltd, China) to represent free interstitial fluid and a large MW tracer fluorescein isothiocyanate dextran (FITC-dextran, MW is 70,000) (Sigma-Aldrich, USA) to represent lymphatic fluid. [11] These tracers allow us to visualize the effluent pathways which collect fluid from subcutaneous interstitial spaces of an acupuncture point.

Considering the acupuncture point of Taixi (KI3) was well studied in our previous studies and pertains to the Kidney Meridian of Foot-Shaoyin located posterior to the medial malleolus, halfway between the Achilles-tendon and the side of the left and/or right ankle-bone, [12] which is able to be precisely located in peripheral microcirculation of both humans and rabbits, it is investigated here to visualize the longest course of draining pathways in hind limbs, intra-abdominal and intra-thoracic cavities of rabbits.

## Materials and Methods

### Subjects

The study is conducted at Beijing Hospital between January 2009 and March 2012. 85 healthy white rabbits (body weight 2–3 kg) are randomly assigned to 17 groups (5 rabbits per group). Study protocol approves by the animal ethics committee of Institute of People’s Hospital of Peking University (No.201170) and China Academy of Chinese Medical Science (No.090115). Anesthesia (pentobarbital at 25–30 mg/kg/hour) is administered intravenously before each of the following experiments. [13] The tracer solution is injected subcutaneously and contained 2% lidocaine (accounting for one third of the total volume) for local anesthesia. Each subject is sacrificed via an overdose of pentobarbital.

### MRI Imaging Method

The imaging is performed via an Achieva 3.0T TX scanner (Philips Electronics, China) located within our hospital. The images are obtained using a 3D T1-weighted fast field echo (FFE) sequence with an 8-channel phased-array head surface coil. Scanning parameters are adjusted in order to obtain a higher spatial resolution. Acquisition time for each sequence is approximately 4–6 minutes. A total of 30 subjects are chosen to receive routine, whole-body MR angiography on a different day than subcutaneous injection. This is done by intravenous injection of Gd-DTPA via the auricular vein (Gd-DTPA dose of 0.1 ml/kg). Raw data are analyzed at the extended MR WorkSpace station with multiplanar reconstruction (MPR) and maximum intensity projection reconstructions (MIPs).

### Method of Determining Transport Speed of Tracer using Gd-DTPA

Each of the 85 rabbits is subcutaneously injected with Gd-DTPA (0.5–1 mL, diluted concentration of 156 mg/mL) into the acupuncture point of right KI3 at a depth of 1–3 mm with 1 ml syringe (B. Braun Medical, Shanghai) on the first day, the left KI3 on the second day, and both the right and left KI3 simultaneously on the third day. Each of the subjects is dynamically scanned approximately 45–60 minutes after injection via repeated 3D T1-weighted fast field echo (FFE) sequences. During the unilateral injection of the right KI3 of 15 rabbits and left KI3 of another 15 rabbits, 2–3 minutes worth of rapid coronal or sagittal scans, each of which takes about 17 seconds, are performed and followed by high resolution FFE scans. The distance, as determined by distance along a curve, is calculated using the reconstructed images obtained for 40 subjects. This is used to determine the tracer’s transport speed originating from KI3.

### Method of Determining Transport Speed of Tracer using Fluorescein Sodium

**Figure 1:**
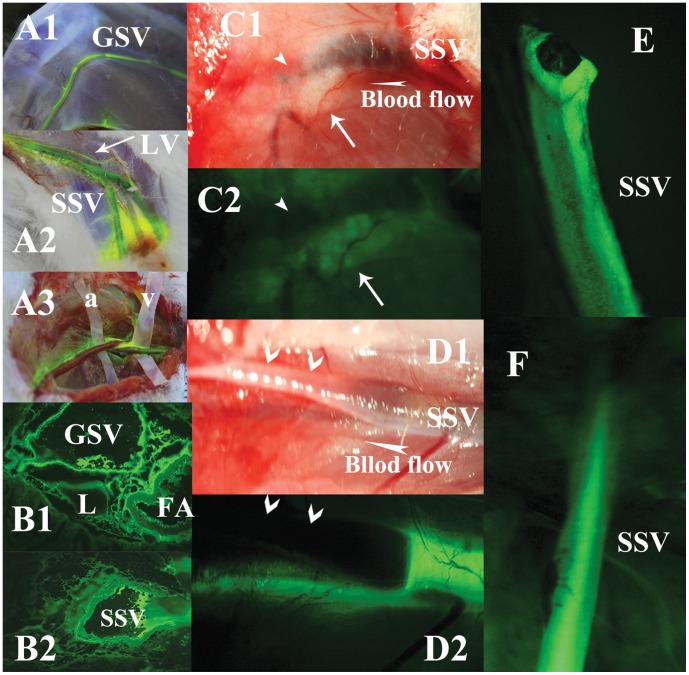
Illustrations of the perivenous tissues, but not artery or intravenous blood, in lower limbs stained by fluorescein from KI3. The vessels walls of GSV (**A1** in the lateral knee), SSV (**A2** under the level of popliteal fossa) and a near lymphatic collecting vessel (LV) are stained by fluorescein from KI3, observed in a rabbit of group I. **A3** shows venous walls (on plastic spacer v), but not of arteries (on plastic spacer a), are stained in anther rabbit in group I. Observed in group XVI, **B1** shows fluorescein accumulates in the interstitial spaces among GSV, FA and LV. **B2** shows strong fluorescein around SSV. In group XIII, **C1** shows the proximal end of SSV is cut off and ligated (arrowhead points at the broken and ligated end of the SSV, white arrow points at the perivenous loose connective tissues remained due to vasoconstriction after the inside vein is cut off). **C2** shows the perivenous tissues are still stained by fluorescein from KI3 even no inside blood vessel. **D1** in group VIII shows a segment of SSV, the surrounding tissues on its walls are stripped off and exposed in the air (dried). **E2** shows the segmental, unstripped and wet SSV is stained by fluorescein from KI3, Meanwhile, the perivenous loose connective tissues beneath SSV are stained as well. **E** shows the entire venous walls with broken end are stained by fluorescein from KI3 clearly, isolated from a subject of group II, in contrast to the intravascular blood that is NOT stained. But **F** shows the intravascular blood in SSV in group XI is stained significantly via intravenous injection.

5 rabbits in group I are subcutaneously injected with 0.2∼1 mL of fluorescein sodium (diluted concentration was 10 g/L by natural saline) in the right KI3 (Figs. 1A1–1A2). An additional 5 rabbits in group II are injected in the left KI3 and 5 rabbits in group III in both the right and left KI3. This is performed at a depth of 1–3 mm, as is also done in the above Gd-DTPA experiments. To observe the flow of fluorescein sodium, a total of 6 rabbits, two rabbits from groups I, II, III are randomly chosen. Incisions are made to form a flap revealing the Greater Saphenous Vein (GSV) or Small Saphenous Vein (SSV) under the facial plane and plastic spacers (constructed from plastic medical gloves) are wrapped around the vessel walls of the artery and vein to isolate the vessels from the surrounding tissue (Fig. 1A3). The flap is then sewn prior to the subcutaneous injections. The rabbits are then sacrificed 40–60 minutes after injection. The hind limbs and intra-abdominal and intra-thoracic cavities of the rabbits are then dissected layer by layer. Any tissue or organs stained by fluorescein are photographed by a digital camera under blue-violet and/or natural light or stereo fluorescence microscope (Nikon SMZ1000, Japan), or sampled for histological studies.

In order to measure the transport speed of fluorescein sodium originating from KI3, 5 rabbits of group IV have a 1–2 cm^2^ are of skin dissected at a position 10 cm away from KI3 to reveal both the GSV and SSV. The exposed veins are dynamically photographed every 5 seconds immediately after injecting fluorescein sodium into KI3. Individual calculated transport speeds are used to determine a mean transport speed.

### Method of Vascular Isolation and Interruption of Specialized Pathways

Within group V, the GSV of the medial thigh is studied in 3 rabbits and the SSV in lateral thigh in another 2 rabbits. Skin above the veins is dissected to expose the GSV and SSV under the facial plane. Small incisions are made to remove a 1 cm length of fascia totally along the walls of the exposed GSV and SSV. Surgical sutures or a 1 cm long plastic spacer are then very carefully inserted into the space beneath the vein to isolate the vessel from the surrounding tissues, while ensuring that no blood flow is compromised and the loose connective tissue of exposed adventitia keeps open to air. Each subject is then immediately injected with Gd-DTPA into the KI3 and scanned for approximately 30–40 minutes using sequential FFE images. To ensure patency of the veins after surgical isolation, 2 mL of Gd-DTPA is then injected intravenously into the auricular vein in the same subjects and sequential FFE images are again taken.

**Figure 2:**
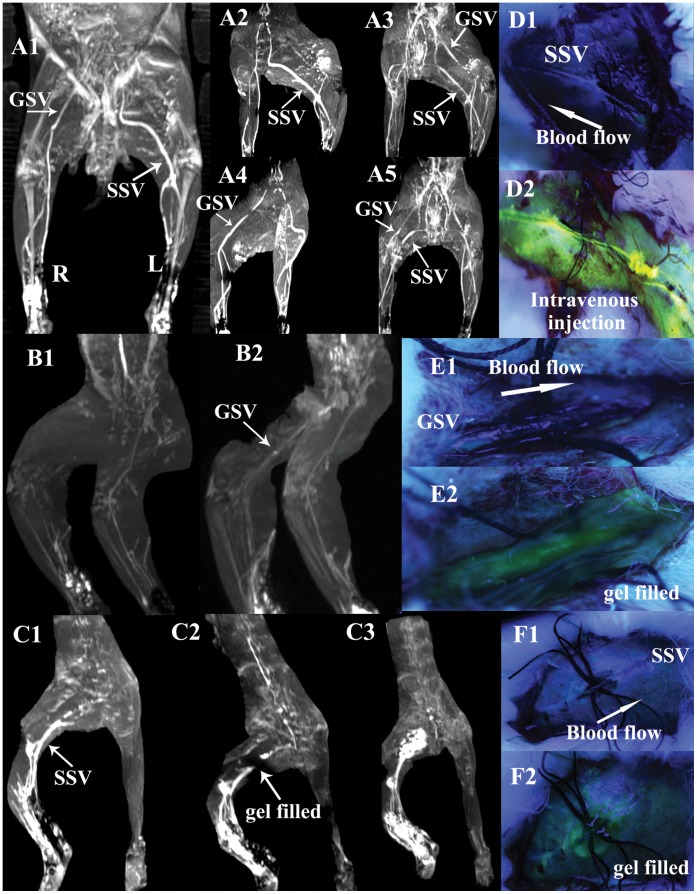
Illustrations of the blockade of the perivenous pathways along GSV or SSV by circular incision and planted sutures, and then restored by transonic gel. **A1–5** show circular incision and planted sutures are performed on segments of left GSV in the medialis knee and right SSV in the lateral thigh in a rabbit of group V. Frontal view (**A**1) and modified lateral views (**A2**, **A4**) show the absence of left “GSV” and right “SSV” scanned at 7 min after KI3 injection in contrast to the intact right “GSV” and left “SSV”. **A3**, **A5**, Two views of lower limbs by angiography through intravenous injection via auricular vein, show the interior vascular lumen of either left GSV or right SSV is not blocked by the operations and keep unobstructed actually. **D1** shows the perivenous transport of fluorescein sodium from KI3 is blocked by circular incision and planted sutures on SSV in a rabbit of group VI. **D2** shows the vascular lumen is actually not obstructed by injecting fluorescein into the vein. **B1** shows the blockade in “GSV”, **C1** in “SSV” of two rabbits in group VII, **E1** in “GSV”, **F1** in “SSV” of another two rabbits of group VIII. After filling transonic gel into the incision sites (showed in **E2**, **F2**), the centripetal transport of Gd-DTPA or fluorescein sodium from KI3 through perivenous tissues is restored. The entire downstream “GSV or SSV” are visualized again (showed in **B2**, **C2**, **E2** and **F2**) (the details of C2 showed in Movie.2). Even the providing-gel is enhanced or stained in a period of time after injection (showed in **C3**, **E2** and **F2**) (the details of C3 showed in Movie.3).

The same procedure is performed in 5 rabbits in group VI, this time using 0.2–0.8 mL of fluorescein sodium injected subcutaneously into the KI3 (Fig. 2D1). Photographs are taken using blue-violet light at two minutes intervals for a total of 30–40 minutes. 0.5 mL fluorescein sodium is then injected intravenously into the isolated vein upstream to confirm patency of the lumen.

### Method to Assess Restoration of Pathways using Transonic Gel or Physiological Saline

The same surgical procedure is again performed in group VII, the exposed adventitia of a segment of saphenous veins has not been enhanced by the small amount of subcutaneous Gd-DTPA from KI3 by the same FFE sequences (Figs. 2B1, 2C1). After the blocked area is clearly visualized, a small amount of transonic gel or saline is then filled around the vessel walls of the exposed segment. Immediately after this is done, MRI imaging is performed for 40–50 minutes to observe whether the tracers could pass through the isolated area using transonic gel (Aquasonic 100, Parker Laboratories, Inc. USA) or saline.

This is repeated in 5 rabbits of group VIII. The region in which the pathway is blocked is clearly photographed after injecting 0.2–0.8 mL fluorescein sodium into KI3 (Figs. 2E1, 2F1). Immediately after the gel or saline is filled around the vessel walls, photographs are taken at 1 minute intervals for another 30 minutes to observe whether the interrupted pathways could be restored using transonic gel or saline.

### Method of Visualizing the Lymphatic Vessels using FITC-dextran

In order to visualize the lymphatic vessels, 0.5∼1 mL lymphatropic FITC-dextran (dilated concentration was 15 g/L) is injected subcutaneously with a depth of about 1–3 mm into the right KI3 of 3 rabbits and the left KI3 of another 2 rabbits in group IX. 30–40 minutes after the injection, the subjects are sacrificed and the stained tissues are photographed.

**Figure 3:**
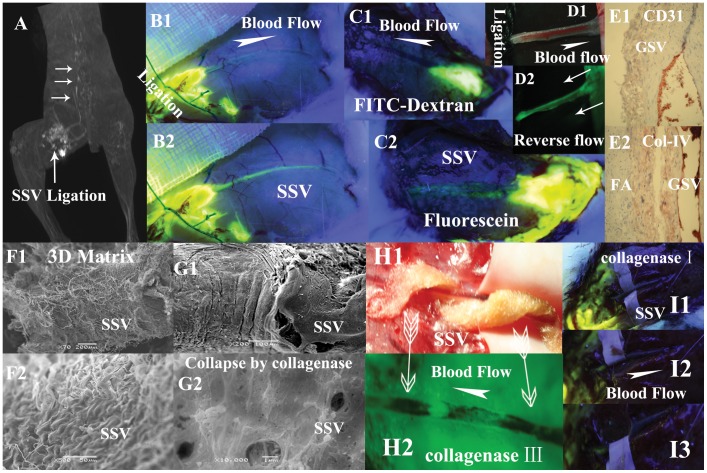
Illustrations of the flow properties of the perivenous pathways exhibited in different groups. **A** (a subject in group XII) and **B** (a subject in group XIII) show the tracers through the perivenous pathways are not due to intravenous blood flow. After the obstruction of a segmental SSV plus its 10 mm surrounding tissues ligated by the sutures, the injected Gd-DTPA in **A** and fluorescein sodium in **B** into the proximal side of the ligation can be transported centripetally along the downstream SSV and vertebral vein pointed at by three white arrows. The circle seen in **A** is water sac due to the injection. **B1**, **B2** are photographed at 5 minutes and 25 minutes respectively after the injection. **C1** shows a small amount of FITC-dextran is injected into the perivenous tissues of SSV above the level of popliteal fossa in a subject of group X and photographed at 30 minutes after injection. There are no fluorescent signals found along the vessel walls of the entire length of downstream SSV. **C2** shows (photographed at 15 minutes after injection) the perivenous pathway is stained by injected fluorescein sodium into the same position of the same subject. **D1** shows the upstream site of a segmental SSV is ligated by suture in group XIII. **D2** shows the fluorescein sodium in the downstream site is pushed backwards by repeated massage performance (two white arrows point at the centrifugal pressure direction) and has stained the perivenous pathway reversely. Inmmunostaining results in **E1** (×40, CD31)**,** **E2** (×20, collagen IV) show no appearances of numerous ring-like groupings or linear structures within the venous adventitia and its surrounding loose connective tissues. **F1** (×200),**F2** (×500) show porous fiber-bed on adventitia of SSV. **G1** (×200), **G2** (×10000) show adventitial porous fiber-bed, collapsed, on a segment of SSV hydrolyzed by collagenase type IV. **H1** shows a cotton ribbon full of 10% collagenase type III spirals round the venous vessel. **H2** demonstrates fluorescein sodium from KI3 is able to bypass the etched gap, be transported along the spiral path and has stained the unetched wall and the downstream venous walls (two arrows point at the positions covered by the cotton ribbon). **I1** shows no fluorescein signals on SSV after hydrolyzed by collagenase type I even large amount of fluorescein are injected into the upstream in group XIV. The SSV is drawn aside by spacer to show no fluorescein signals found in surrounding tissues in **I2**, **I3**.

Immediately after this, a 5–6 cm skin incision is made along the course of the SSV above the level of popliteal fossa. 0.2 mL FITC-dextran is carefully injected into the space between the overlying fascia and the wall of the SSV. The exposed vein is dynamically photographed for 30 minutes (Fig. 3C1). Afterwards, 0.2 mL fluorescein sodium is then injected into the same space and the site is photographed for another 30 minutes (Fig. 3C2).

### Method to Compare the Pathway of Subcutaneous Versus Intravenous Injections

In order to compare the results of subcutaneous injection with those of intravenous injection, 1 mL fluorescein sodium is intravenously injected into the auricular vein of each rabbit in group XI. All 5 subjects are sacrificed after 15–20 minutes and then stained tissues are photographed and sampled.

### Method to Assess the Effect of Vascular Isolation on Tracer Transport

In group XII, a segment of the SSV located in posterior thigh is chosen to be completely ligated together with its surrounding 5–10 mm muscle tissues using surgical sutures. 0.5–1 mL Gd-DTPA is then injected beneath the fascia proximal to the ligation site, in order to observe whether the tracer could still be transported in the specialized pathways via repeated MRI scanning for 30–40 minutes.

In another 5 rabbits in group XIII, the same procedure is performed in 2 subjects using 0.2 mL fluorescein sodium injected into the proximal ligation side (Figs. 3B1–3B2). After a segmental SSV is isolated from its surrounding tissues in another 3 rabbits, the upstream site of the exposed vein is ligated and 0.2 mL fluorescein sodium is injected into the downstream site (Fig. 3D1). And then repeated massage is performed right on the un-exposed vein and towards the opposite of blood flow direction, which can generate an extra centrifugal pressure (Fig. 3D2).

### Method of Assessing Pathway after Acupuncture

A 2–3 cm^2^ incision is performed along the GSV or SSV of subjects in group XIV, and using a steel acupuncture needle, repeated acupuncture is performed on the loose connective tissue closest to the veins while the veins themselves remained intact. Afterwards, subjects are injected with 0.6 mL mixed tracers (0.3 mL Gd-DTPA and 0.3 mL fluorescein sodium) into KI3 and scanned by MRI or photographed respectively.

### Method to Assess the Injection Points and the Specialized Pathways

The two low MW tracers, Gd-DTPA and fluorescein sodium, have strong concordance in visualizing the specialized pathways. Thus, we combine them. In each subject within group XV, three points 2 cm, 4 cm and 8 cm curve distance away from KI3 located above the branches of the saphenous vein are separately injected with 0.6 mL mixed tracers. Another four points 0.3 cm, 1 cm, 1.5 cm and 2 vcm perpendicular to the long axis of the saphenous vein is also injected with 0.1–1 mL mixed tracers. Every injection point is scanned by MRI one by one on separate days. When a specific pathway is visualized clearly by Gd-DTPA, the area is dissected and observed under blue-violet light.

### Method to Assess the Effect of Collagenase on the Specialized Pathways

For each subject in group XIV, a 1 cm long plastic spacer is wrapped around an exposed segment of the GSV or SSV two weeks after the above experiments. Drops of 10% solution of collagenase type I, III, IV or V are administered at 1–2 minute intervals to keep the vessel walls lubricated for a total of 15–20 minutes (Fig. 3I1). [14] Or a cotton ribbon full of the 10% collagenase solution of either of type I, III, IV or V spirals the venous vessels more than one round at least (Fig. 3H1).

The effects of collagenase type I, III, IV or V on the SSV and GSV are investigated using 5 subjects. Multiple segments of SSV and GSV are to determine the effects of collagenase on these specialized pathways: 3 segments of GSV and 3 segments of SSV for collagenase type I, 2 segments of GSV and 2 segments of SSV for collagenase type III, 2 segments of GSV and 2 segments of SSV for collagenase type IV, and 3 segments of GSV and 3 segments of SSV for collagenase type V. Afterwards, 0.2∼0.4 mL fluorescein sodium is injected into KI3 to observe whether the exposed segments are stained (drops of saline are administered to keep the vessel lubricated during the observation period). The venous segments hydrolyzed by collagenase as well as the preserved portions of the vein are stored in 4°C 2.5% glutaraldehyde solution in preparation for scanning electron microscope (SEM) studies.

### Method for Preparing Pathologic Specimens

In group XVI, all 5 rabbits are sacrificed within 10–20 minutes after the subcutaneous injection of 0.5–1 mL fluorescein sodium into the left and right KI3. Using a microtome (YD-202, Zhejiang-Jinghua, China), 5 cm longitudinal segments of GSV or SSV as well as the surrounding muscle are sampled and frozen or stored in 15% formaldehyde solution for histological studies or SEM. Frozen sections of 10 µm thickness are studied under a fluorescein microscope. Stained tissues or organs of the intra-abdominal and intra-thoracic cavities are subsequently photographed.

### Method for the Detection of Pericardial Fluid by Echocardiography

In group XVII, immediately after a total of 2–5 mL fluorescein sodium is injected into both sides of KI3 in 3 rabbits, and 5 mL intravenously via ear vein in another 2 rabbits, echocardiography is performed dynamically to detect the changes of pericardial fluid. The rabbits are sacrificed and their thoracic cavities are opened after the detection.

### Tissue Sampling for Histological & Immunohistological Studies

After Haematoxylin & Eosin (HE) staining and immunostaining [15] of the antibodies against CD31 (JC/70A, Abcam, MA, USA) and collagen IV (4595R, Abcam, MA, USA), any fluorescently stained tissue samples are studied via an optical microscope. Chinese ink is used to permanently locate the exact position of fluorescence in the tissue samples.

## Results

### Vasculature in Hind Limbs Visualized by Low MW Tracers

When each subject is injected subcutaneously at a depth of 1–2 mm with either Gd-DTPA or fluorescein sodium into KI3, there is not only regional diffusion around the injection site but also centripetally-transported pathways originating from KI3 that begin to gradually appear in the injected hind limb approximately 1.5–2 minutes after the injection. This becomes increasingly visible after 3–5 minutes (Figure S1A–S1D3, Video S1).

The pathways visualized by Gd-DTPA remain strongly visible from 5–40 minutes and begin to disappear after 40–50 minutes. The disappearance rate depends on the dosage of magnetic contrast injected (slower in the 1 mL dose than the 0.5 mL dose). The pathways are gradually visualized by fluorescein sodium and remain strongly visible during both the observation period and subsequent anatomical sampling procedures.

In addition, the pathways displayed by Gd-DTPA are the same pathways visualized by the fluorescein sodium when comparing the reconstructed images by MRI with the photos by digital camera (Figs. 1A1–1A2, Figure S1B1–S1D3). These illustrated pathways originating from KI3 coincide with the GSV, SSV, and their respective main branches located in the hind limbs as well as the inferior vena cava (IVC), vertebral veins (VV), aorta (AO) and pulmonary arteries (PA) in the intra-abdominal and intra-thoracic cavities. This is confirmed by 3D contrast-enhanced MR angiography (ce-MRA) by intravenously injecting Gd-DTPA into the auricular vein (Figure S1F1-S1F6, S2A-S2G3). The popliteal lymph nodes and their related afferent and efferent lymphatic vessels are also visualized by Gd-DTPA (Figure S1E1-S1E3) or fluorescein sodium injected at a depth of 2–3 mm.

When a small amount of Gd-DTPA or fluorescein sodium is injected into KI3 unilaterally, strongly enhanced or stained tissues surrounding the veins are only seen in the injected hind leg of groups I and II. In contrast, the opposite (non-injected) hind leg does not exhibit any strong magnetic or fluorescent signals in the tissues surrounding the vessel wall during the observation period (Figure S1B1-S1B2). Of note, the magnetic signals in the bladder begin to gradually appear after 5–10 minutes and becomes increasingly stronger after 15–20 minutes (Figure S1B2, S1C3, S1D3).

### Centripetally Transport Speed Measured by MRI & Fluorescein Photography

Via coronal or sagittal MRI scans, the approximate transport speed of the tracer through the pathways originating from KI3 in either the right or left hind limbs of 30 subjects is measured respectively. The mean migration speed measured by this method is 0.2–1 cm/sec. By digitally photographing 5 rabbits of group IV while injecting fluorescein sodium into KI3, the approximate speed of the fluorescent agent through the pathways originating from KI3 is calculated at 0.1–2 cm/sec.

### Blockade of the Pathways by Isolation of either the GSV or SSV

MRI or fluorescein photography performs in group V and VI with the GSV and SSV dissection area exposed demonstrate that the transport of Gd-DTPA and fluorescein sodium originating from KI3 along GSV or SSV is completely blocked by the isolation procedure performed without compromising blood flow in the vein itself (Figs. 2A1, 2A2, 2A4, 2B1, 2C1, 2D1, 2E1, 2E1, 2F1). This is confirmed by intravenously injecting Gd-DTPA into the auricular vein (Figs. 2A3, 2A5) and directing injecting fluorescein sodium upstream from the isolated portion of the vein (Fig. 2D2).

### Restoration of the Specialized Pathways by Filling Transonic Gel, Saline around Isolated Vein

When transonic gel or saline is filled around the isolated vein prior to injection of the low MW tracer into KI3, the tracer’s transport through the specialized pathways is restored (Figs. 2B2, 2C2, 2E2, 2F2, Video S2). The transonic gel itself around the incision is enhanced by Gd-DTPA in group VII (Fig. 2C3, Video S3) or stained by fluorescence in group VIII (Figs. 2E2, 2F2) respectively.

### Visualization of Lymphatic Vessels Originating from KI3 using FITC-dextran

In group IX, all collecting lymphatic vessels originating from KI3, including the popliteal lymphatic nodes and their afferent and efferent lymphatic vessels, are clearly visualized in the hind limbs under blue-violet light (Figure S3B2-S3B3). However, there is no fluorescent signal of the GSV’s or SSV’s vessel walls or their main branches when FITC-dextran is used (Figure S3B2-S3B3). Moreover, there are no collecting lymphatic vessels originating from KI3 visualized aside from the part of SSV superior to the popliteal fossa (Fig. S3B3). This coincides with histological studies as well.

### Transport of FITC-dextran Versus Fluorescein Sodium in the Specialized Pathways

In group X, 30 minutes after injection of FITC-dextran, the large MW fluorescent tracer is still located at the injection site with no fluorescent signal found at any point downstream from the injection site of the SSV (Fig. 3C1). When a small amount of fluorescein sodium is injected into the same position of the same subject, a fluorescent signal gradually emerges along the vessel wall of the SSV downstream from the injection site (Fig. 3C2).

### The Distribution of Intravenous Fluorescein Sodium in Blood Circulation

In group XI, intravenous fluorescence quickly stains all tissues of the whole body. The fluorescent signal of superficial tissues is averaged and dispersed. No subject has any signs of fluorescein accumulation around the vessels wall of the GSV (Figure S3C2) or SSV (Figure S3C3). In the abdominal and thoracic cavity, the entire length of the small intestinal wall (Figure S3C1) and all the pulmonary veins are stained by fluorescein sodium. Strong fluorescent signals are also found in all surfaces of the heart, including the coronary groove, anterior and posterior inter-ventricular grooves, left and right auricles, all the myocardium and no extra pericardial fluid (Figure S4B1-S4B3). Part of the thoracic duct adjacent to the thoracic aorta is visualized. However, the lymphatic trunks beside IVC are not visualized by intravenous tracer (Figure S4B4).

### Blood Flow in Veins Taking no Effects on the Tracers’ Transport along the Specialized Pathways

In group XII and group XIII, despite ligation of the SSV and the surrounding 5–10 mm muscle tissue with surgical sutures, the low MW tracers in the loose connective tissue proximal to the ligation site is still able to be drained centripetally along the downstream vein (Figs. 3A, 3B1–3B2), albeit with a much slower transport speed. By the extra centrifugal pressure, the downstream fluorescein sodium is pushed backwards to have stained the upstream of the perivenous pathways (Fig. 3D2).

### “Puncture-resistant” Specialized Pathways

Even after repeated puncturing using a steel needle at the acupuncture site, the specialized pathways are still able to transport the low MW tracers centripetally. This is confirmed by either MRI or fluorescein photography in group XIV.

### The Effect of Collagenase Type I, III, IV and V on Transport of Fluorescein Sodium

Hydrolyzed by either of collagenase type I, III, IV or V in group XIV, none of the entire segmental saphenous veins (Figs. 3I1–3I3) is stained. However, in the subjects etched by spiral cotton (Fig. 3H1), fluorescein sodium from KI3 is able to bypass the etched gap to stain the other un-etched spiral ribbon forming a fluorescent spiral line around vessel wall, like a barber pole (Fig. 3H2).

### The Relation between the other Injection Points and the Specialized Pathways

Using MRI or fluorescein photography in group XV, the tracers in each of three points on top of saphenous veins would emerge the downstream specific pathways directly. The tracer of 0.2 mL, in the point that is injected at a 0.3 cm perpendicular distance away from the long axis of the saphenous vein, would also emerge the specialized pathways. However, the tracers injected at a distance of 1 cm, 1.5 cm, 2 cm perpendicular to saphenous veins are only able to converge into the specialized pathways at larger injection doses. There are no additional specialized pathways found beyond those visualized along the GSV, SSV and their main branches.

### The Unique Distribution of Fluorescein Sodium Originating from KI3 within the Abdominal and Thoracic Cavities

The subjects in group XVI are sacrificed 10–20 minutes after subcutaneous injection of KI3 to observe the staining pattern of fluorescein sodium within the abdominal and thoracic cavities. In contrast to the findings of intravenous fluorescent signals seen throughout entire length of the small intestine, the pulmonary vasculature, and the whole heart surfaces without extra pericardial fluid, only segments of the small intestine (Figure S3A1), the right superior pulmonary veins (Figure S3A3) are stained by fluorescein from KI3. Particularly in groups XVI and XVII, pericardial fluid is found along the coronary groove, anterior and posterior inter-ventricular grooves, meanwhile the fluorescein sodium strongly accumulates within them as well as the left and right auricles in contrast to other parts of the heart (Figure S4A1-S4A3, S4A5). In addition, the tissues covering IVC (including adipose tissues) are also stained fluorescently (Figure S4A4-S4A9, S4A11), which seems stained by fluorescein from KI3, but it is difficult to be distinguished from the results of intravenous injection by the presented imaging methods. Further studies are needed.

There is also no fluorescent signal found in all of the small intestines, pulmonary veins and the heart surfaces when injecting FITC-dextran into KI3 of group IX (Figure S3B1, S4C1-S4C2), except the lymphatic trunks in the vicinity of IVC (Figure S4C3).

### Results of 2–5 mL Fluorescein Sodium Injection into KI3 by Dynamical Echocardiography and Fluorescence Imaging

In the 3 subjects of injection into KI3, pericardial fluid begins to gradually appear in approximately 20–30 minutes after the injection. This becomes increasingly visible after 40–60 minutes. When thoracic and abdominal cavities opens, the results of the subjects injected with 2 mL are similar with those of group XVI. The stained pericardial fluid is mainly distributed along the three grooves. In those with 5 mL injection, a large amount of pericardial fluid is found (Figure S4A10). In addition, much longer small intestine tube is stained by 2–5 mL fluorescein sodium from KI3 (Figure S3A2) in contrast to those of group XVI. But all pulmonary veins are stained together by 2–5 mL injection. By contrast, in those 2 subjects with 5 mL intravenous injection, there is no extra fluid found in pericardium during a 90 minutes observation.

### Histological & Immunohistological Results using Optical Microscopy

Arterial and lymphatic vessels are clearly visualized running along the GSV in the medial aspect of the subjects’ thigh, but they are not observed along the SSVs of the subjects’ lateral thigh. Aside from lymphatic vasculature, no longitudinal tubal structures or abundant capillary networks are identified in either venous adventitia or the perivenous loose connective tissues in close proximity to great and small saphenous veins sampled from ankles to groins by means of HE staining and immunostaining of anti-CD31 (Fig. 3E1) and anti-collagen IV (Fig. 3E2). The unique tissue surrounding the venous and/or arterial vessels walls is loose connective tissue composed of adipose and collagen.

### Results by Fluorescein Microscopy or Stereo Fluorescence Microscope

5 subjects of group XVI are sacrificed 10–20 minutes after subcutaneous injection of fluorescein sodium into KI3. As observed by fluorescein microscopy, the tracer stains the spaces among the GSV and FA in the medial thigh (Fig. 1B1), as well as the tissues surrounding SSV in the lateral thigh (Fig. 1B2). There is no obvious membrane or tissue border to suggest a vascular space. Observed by stereo fluorescence microscope, the fluorescein from KI3 stains both of the perivenous loose connective tissues and venous adventitia (Figs. 1C1-1C2, 1D1-1D2) but not intravascular blood (Fig. 1E).

### Histology of Stained Tissue along the Specialized Pathways using SEM

Bundles of collagen fibrils arranged in a crisscross pattern embedded by porous mesh are found within the saphenous venous walls (Figs. 3F1-3F2) and arterial adventitia. By contrast, for those in group XIV who are hydrolyzed by either of collagenase type I, III, IV and V, the porous collagenous structures on vessels walls collapse (Figs. 3G1-3G2). From ankle to groin in each sample of group XVI, there are no unknown longitudinal tubal structures or abundant capillary networks found in the venous adventitia and perivenous loose connective tissues aside from adipose tissue and collagen fibers by SEM, HE and immunohistological staining (Figs. 3E1-3E2).

## Discussion

The extracellular tracers, Gd-DTPA and fluorescein sodium injected in the subcutaneous loose connective tissue of KI3, would mix with free interstitial fluid and enter into blood and lymphatic vascular trees, such as postcapillary venules, collecting venules, saphenous veins and adjacent lymphatics, besides local diffusion (Figure S1C1, Video S1). In addition to the conventional “tree-like” drainage pathways, it is demonstrated in this study that either paramagnetic or fluorescein sodium has been transported through the venous adventitia, but not through intravascular blood (Fig. 1F), of the entire length of the GSV, SSV and their branches as well as their surrounding loose connective tissues (Figs. 1A1-1A2, Figure S1D1-S1D3), except for the accompanying arteries (Fig. 1A3).

It might be thought that the low MW tracers were transported by interconnected network of vasa vasorum within the walls of blood vessels. It was reported, for vasa lumen diameters >40 µm in diameter, that the hierarchical architecture of vasa vasorum within porcine coronary arteries is “tree-like” without network or plexus. [16] However, for the smaller vasa vasorum that could not be visualized easily by imaging technique or immunohistological staining of CD31 and collagen IV (Figs. 3E1-3E2), have they formed network?

There are only two sources of vasa vasorum, adjacent arteries or veins. If it were arterial, the tracer within the wall of the GSV and SSV would have originated from the heart. It, therefore, should have equally transported into both the right and left femoral artery, thereby staining both the GSV and SSV. However, this is not observed in group I or II, in which the opposite (non-injected) hind leg does not exhibit any strong magnetic or fluorescent signals in the tissues surrounding the vessels walls (Figure S1B1-S1B2). In addition, there is no sign of increasing fluorescein accumulation around the vessel wall of the GSV (Figure S3C2) or SSV (Figure S3C3) after intravenous injection of the auricular vein in any subject within group XI. These results argue against the possibility that the tracers originated from arterial vasa vasorum. Additionally, the calculated transporting speed is not consistent with that of venous vasa vasorum.

Furthermore, our findings, that fluorescein sodium but not FITC-dextran could be transported through these visualized pathways (Figs. 3C1-3C2), also denies the possibility of an extensive network of lymphatic capillaries being existed in the venous walls and their surrounding loose connective tissues because a lymphatic capillary network should have the ability to transport the large MW tracer FITC-dextran due to its physiological characteristics.

Thus, the drainage through these perivenous pathways is similar to that of non-vascular interstitial fluid transport studied extensively in brain, tumor, musculoskeletal tissues and soft connective tissues (e.g. tendons or ligaments), [17]–[23] which is generally due to two patterns, perivascular spaces acting as conduits and extracellular matrix (ECM) as porous medium.

Perivascular spaces are a conduit space between a single layer of pia mater and each vessel of arteries and veins of brain, through which cerebrospinal fluid flow with the possible mechanisms of convective transport and “perivascular pumping” driven by pulsations of the vessel walls. [17], [18] In contrast to current findings (Figs. 1B1-1B2, 3F1-3F2), the visualized perivenous pathways, consisting of general loose connective tissues without a tunnel covered by any well-defined boundary membranes, should not be a perivascular-spaces-like channel. [8].

On the other hand, the ECM of fibrous tissues, such as brain, solid tumors, soft connective tissues, adipose tissues and many other biological tissues, can be considered as porous medium composed of collagen fibers scaffolds with proteoglycans, and other molecules contents, [19]–[25] through which is also capable to provide permeability paths (characterized by continuous porous network) for fluid to move. It is essential to remain the integrity of the three-dimensional (3D) scaffolds of porous medium to maintain the transporting function.

Demonstrated by SEM in this study, the visualized perivenous pathways appears to consist of collagen-rich loose connective tissues with porous fiber-bed (Figs. 3F1-3F2) in accordance with the collagenous microstructures of general loose connective tissues. When a small amount of water solution containing either collagenase type [26] I, III, IV or V is added on the adventitia of a segment of saphenous veins, the centripetal transport of fluorescein sodium through the perivenous pathways is interrupted (Figs. 3H1-3H2, 3I1-3I3). In addition, the findings by SEM clearly show that the 3D porous collagenous scaffold on vessels walls collapsed by collagenases (Figs. 3G1-3G2). These results, together with other previous studies, [19] indicate a stabilizing collagen matrix is primary to attain the permeability for free fluid transport. The reason to only study the adventitia is the difficulties of isolating the surrounding loose connective tissues from adventitia as well as no differences between them according to histological knowledge.

Usually, free interstitial fluid is considered to be mainly entrapped within the gel-like substances of most tissues after exchanged by filtration or diffusion from vasculature unless extra pressure or concentration gradients are performed. [27]–[29] However, neither the pressure nor concentration gradients caused by a small amount of tracer fluid injection are able to explain the penetration into perivenous tissues farther away from KI3, especially while the visualized pathways remain exposed in the air without extra pressure.

Moreover, a porous medium (or a solid matrix) can be characterized as a two-phase immiscible mixture. [30] One phase is an incompressible solid with porous-permeability, mainly collagen fibers and proteoglycan. The other is an interstitial fluid phase, primarily incompressible water. In present results, when the adventitia is exposed to air and free adventitial water mainly evaporated, [31] the centripetal fluid flow through the pathways is interrupted, until free water is resupplied by adding transonic gel or natural saline (Figs. 2B1-2B2, 2C1-2C3, 2E1-2E2, 2F1-2F2). These findings give more evidences that not only free residual water exists but also the perivenous pathways are an open path without membranes and boundaries to protect the water from evaporation, which also indicate the velocity of free water through the pathways is much slower, especially than that of evaporation.

Therefore, the presented phenomena strongly suggest a unique pathway in a specialized loose connective tissue, characterized by water-rich collagenous porous scaffolds, have a high hydraulic conductivity for free interstitial fluid wherein a phase of the collapse of their intact porous fiber-beds would decrease its hydraulic conductivity, the flow pattern of which seems more like fluid flow through numerous micro pores networks but not due to convection in a conduit. [17].

As a high hydraulic conductive porous matrix, the pressure gradients and continuous fluid incoming determine the flow direction. Instead of complicated devices and equipment to measure interstitial hydraulic pressure in tissues [27], [32], [33], it is more convenient to directly observe fluid flow by the help of low MW extracellular imaging agents in vivo. The results in group XIII clearly show the perivenous porous matrix is capable to transport free water centrifugally when extra opposite pressure is performed (Figs. 3D1-3D2). The similar phenomenon was observed in some other specialized tissues, such as mucosal microcirculation of intestines and synovium.[34]–[36] An independent stream of fluid flow through interstitial spaces (i.e. not local capillary filtrate) could be resulted by sustained and continuous fluid absorption.

Further findings show that, into surrounding subcutaneous connective tissues wherever are along saphenous veins, both Gd-DTPA and fluorescein sodium would visualize the downstream venous walls clearly. Within perpendicular distance of 0.3 cm to the visualized pathways, the low MW tracers of 0.1 mL in adjacent loose connective tissue would visualize the pathways too. If the perpendicular distance to the pathways is increased, a larger amount of tracers is necessary to eventually visualize the specialized pathways. In addition, the results in group XII and group XIII show, the Gd-DTPA and fluorescein sodium tracers in the proximal side of the ligated area could still be transported centripetally along the downstream vein (Figs. 3B1–3B2) even no blood or lymphatic flow could pass through the complete ligation, which denies the driving power generates from blood flow. These data indicate that free fluid along the porous pathways could be accumulated to form a relatively closed pathway draining free interstitial fluid centripetally, which may constitute a potential drainage system complementary to vascular circulations.

Some other similar studies also indicated a pathway, distinguishing from blood and lymphatic circulations, is able to transport interstitial fluid, such as tissue fluid flow [37] and BHD system.

The main hypothesis of tissue fluid flow is based on the findings of the parallel nature of capillaries that could lead to directional interstitial fluid flow in the direction of capillaries by numerical simulation, which is different from the current results of HE and immunohistological staining that there is no abundant capillaries distributed within the visualized pathways.

The principal methodological strategies of BHD theory [2], [38] are similar with the present study trying to visualize some specific anatomical structures by injecting various imaging agents into an acupuncture point. Different from the intravascular BHDs, both the previous studies by nuclear tracers [6] and the current study by the fluorescent or magnetic tracers from KI3 clearly show the visualized pathways are not the results of the tracers through the vascular vessels (Fig. 1E) and without the endothelial membrane or tissue border (Figs. 1B1-1B2). Moreover, the findings of spiral flow path (Fig. 3H2) also suggest fluid flow through porous medium but not a longitudinal conduit.

Further findings in intra-abdominal and intra-thoracic cavities show that the general loose connective tissues covering on IVC, (Figure S4A11) are clearly stained by fluorescein from KI3, the results of which are similar with those of intravenous injection (Figure S4B4) and difficult to be distinguished by current imaging methods. However, by carefully compared with the way of intravenous injection that stains all small intestines, pulmonary veins and the surfaces of heart, and of FITC-dextran injection into KI3 that does not stain any intestines, pulmonary veins and the surfaces of heart (except thoracic duct), it is clearly showed that a small amount of subcutaneous fluorescein sodium from KI3 stains only segments of the small intestine (Figure S3A1), the right superior pulmonary veins (Figure S3A3), and increased fluorescein sodium stains longer small intestine tube (not entire length) (Figure S3A2). Particularly, the fluorescein sodium from KI3 strongly accumulates within the coronary groove, anterior and posterior inter-ventricular grooves, left and right auricles, and extra pericardial fluid (Figure S4A1-S4A5), and likely, the fluorescein seems coming from the tissues (including adipose) covering IVC and into the three grooves on heart (Figure S4A6-S4A9). Because of methodological difficulties, what exact structures mediate fluorescein sodium (from KI3) that originates from the connective tissues on IVC into the partial walls of small intestine, pulmonary veins and the surfaces of heart need further studies.

In physiological knowledge, pericardial fluid is secreted by the serous visceral pericardium to lubricate the epicardial surface. Interestingly, a total of 2–5 mL fluorescent natural saline injected into KI3 of both sides could result in pericardial effusion (Video S4-S5), which can increase as the amount of injection water increases, observed by dynamical echocardiography after the injection, in contrast to none by the intravenous injection of the same amount of saline. After sacrificed, it clearly shows that the pericardial fluid is mostly distributed over the three grooves of heart in the subjects with 2 mL injection (Figure S4A1-S4A3). Speculatively, the extra fluid inside pericardial cavity is probably due to the perivenous loose connective tissues draining free fluid along IVC (Figure S4A4-S4A9), though further related studies are needed. These findings may provide another possible mechanism of fluid generation in serous cavity and provoke relevant studies of their potential physiological effects on heart and coronary vascular functions.

As biological porous medium, a systematically-distributed loose ECM with a function of draining free fluid is identified and located in a perivenous loose connective tissue of rabbits, which is only part of the whole loose connective tissues with various tightness in the body. Is there other circulatory loosely-assembled ECM distributed beyond the proximity of the saphenous veins in rabbits? Similar with the characteristic “puncture-resistant” pattern (demonstrated in group XIV), a subcutaneous migration channel previously found in humans is confirmed not venous plexus. What is the relationship of visualized pathways by low MW tracers between the two species? The present evidences are probably clues to eventually reveal the anatomical and histological substance basis of the migration channels in humans.

The initial purpose of this animal experiment is to study the biological phenomenon that a pathway can be visualized by injecting low MW tracers into KI3. It is more proper to say that the specialized pathways come from a perivenous injection point of a rabbit rather than an acupuncture point. However, the panoramic scenario of the perivenous pathways disclosed in present and various previous studies [1], [3]–[8], [39], 40 might suggest a novel systematically-distributed pathway exist in a mammal, which is similar with a description of meridians and collaterals narrated by the Chapter 33 of *Miraculous Pivot* “internally, 12 main meridians connect with the Zang-fu organs, and externally with the joints, limbs and other superficial tissues of the body”, [41] as well as another Tibetan medical literature of *Four Tantras of Tibetan Medicine* “in a view of micro scales, human body is made up by complex networks of numerous different interconnected channels, among which, blood is transported by around 72,000 channels, “Qi” by another 21,600 channels and etc. according to a category based on the interior contents inside the channels.” [42] Of course, more experiments about the visualized non-vascular transport and its relevance with the vascular circulations, the neural hypothesis of meridians [39] and acupuncture’s remote effect [40] are needed.

Above of all, despite lots of questions need further explorations, this study adds substantial insights into a collagenous connective tissue with transport mechanism of fluid flow through porous medium that may offer a bridge to make further researches on ancient medical literatures in the whole world. The displayed connections between peripheral circulation and pericardial fluid, small intestines may be a key to understand additional visceral organs’ functions, nutrition of blood vessel walls and even related therapeutics and pharmacotherapeutics besides the current knowledge about acupuncture’s value in health care. The pattern of non-vascular transport revealed here may provoke great interests on exploring its driving power and mechanism, transport phenomena of various substances through the specialized biological porous medium, and its physiological and pathophysiological functions in living beings.

## Supporting Information

Figure S1 **Illustrations of the pathways in lower limbs of rabbits by MRI.** **A**, Blank view scans before injecting Gd-DTPA. **B**, Two angular views scan at 2 min after injection into right KI3 at 1–2 mm depth. GSV, SSV plus their main branches began to be displayed. **C**, Three angular views scan at 7 min after injection with increasing contrast’s signals. **C1** is modified and shows right GSV, SSV and segments of inferior vena cava (IVC) and vertebral vein (VV). **D**, Scanned at 15 min, the signals in right GSV, SSV, IVC and VV are much more clearly. **B2**, **C3** and **D3** show tracer is collected increasingly in bladder. In the meanwhile, the signals in left GSV and SSV are very weak. **E**, Three angular views of the same subject are injected into right KI3 at 2–3 mm depth and scanned at 15 min from start in another day. Not only right GSV, SSV, IVC and VV but also popliteal lymph node (LN) and segments of afferent and efferent lymphatic collecting vessels (LV) are displayed together. **F**, Different views of angiography in lower limbs of the same subject scan in the other day, which coincide greatly with the pathways coming from KI3.
(TIF)

Figure S2 **Illustrations of the pathways in intra-abdominal and intra-thoracic cavities and lower limbs of a rabbit scanned at 7 min after injecting tracer into right and left KI3 simultaneously.** **A**, **B**, **C**, **D** show clearly IVC, external jugular veins (EJV), VV, pulmonary arteries (PA) and aorta (AO) in different views. **F1**, **F2** show unabridged views of right and left GSV, SSV plus their main branches. **G1**, **G2**, **G3** show different views of GSV, SSV and VV. **E**, Angiography by intravenous injection via auricular vein. IVC, VV, AO and hepatic arteries (HA) are clearly displayed. By comparing with the images of angiography by MRA, the pathways coming from KI3 coincide with the veins and arteries in trunk and lower limbs.
(TIF)

Figure S3 **Illustrations of the differences among different tracers in intra-abdominal and intra-thoracic cavities.** **A1** shows only partial walls of small intestines are stained by a small amount of fluorescein sodium coming from KI3, and a longer intestine tube is stained by higher amount of fluorescein from KI3 showed in **A2**, in contrast to the walls of entire length of small intestines are stained by intravenous fluorescein sodium in **C1**, and no fluorescent signals found on small intestines in **B1** by injecting FITC-dextran into KI3 in group IX. **A3** show fluorescently stained right pulmonary veins in contrast to non-stained left pulmonary veins in **A4** by fluorescein sodium from KI3 in group XVI. Lymphatic vessels (LV) are displayed in the vicinity of GSV in **B2**, SSVs in **B3** (pointed by two white arrows) by FITC-dextran from KI3. **B3** showed there were no collecting lymphatic vessels beside SSV above the level of popliteal fossa (PF). **Note**: **B2, B3** also show there are no any venous walls stained by subcutaneous FITC-dextran injection into KI3. No increasingly accumulated strong fluorescent signals are found in the surrounding loose connective tissues along the entire length of GSV (**C2**) or SSV (**C3**) by intravenous fluorescein sodium into auricular vein in group XI.
(TIF)

Figure S4 **Illustrations of the differences among stained tissues over the heart and beside IVC by different tracers.** Three views of the heart show the fluorescein sodium from KI3 have strongly accumulated and stained some tissues over the heart, including coronary groove in **A1**, a few amount of pericardial fluid in anterior inter-ventricular groove (AIG) and the superficial tissues on the left auricle in **A2**, and right auricle in **A3** in a subject of group XVI. However, the other parts of the heart seem stained by less fluorescent signals in contrast to the strongly stained tissues all over the heart by intravenous fluorescein sodium in **B1**, **B2** and **B3** of group XI. In addition, the surfaces of IVC (**A6**, **A7**) including adipose tissues on it (**A8**, **A9**) and the tissues within coronary groove and posterior inter-ventricular groove (PIG) (**A4**, **A5**) are strongly stained by fluorescein sodium from KI3, which seems fluorescein sodium from KI3 has been transported along IVC and into three grooves to have stained pericardial fluid, speculatively. **A10** shows a larger amount of pericardial fluid in the heart of a subject in group XVII with 5 mL injection into KI3, sacrificed at 90 minutes after the injection and the detection by echocardiography. Few amount of fluorescein sodium originating from KI3 are found over the loose connective tissues on IVC, pointed at by white arrows in **A5**. Only thoracic duct passing through the cardiac base is visualized by FITC-dextran from KI3, showed in **C1**, and no fluorescent signals on IVC in **C2** of group IX. The lymphatic trunks beside IVC are displayed by either fluorescein sodium from KI3 (**A12**) or FITC-dextran from KI3 (**C3**), but not by intravenous fluorescein sodium (**B4**).
(TIF)

Video S1 **Illustration of the specialized perivenous pathways in left hind limb originating from KI3.** The movie is scanned at 5 minutes after the injection at a depth of 1–2 mm. It looks like the GSV and SSV are enhanced by Gd-DTPA. But in fact, the magnetic signals in the pathways are not equally distributed along the entire length of the pathways from ankle to waist, which indicates the Gd-DTPA is transported not through intravenous lumen.
(MOV)

Video S2 **Illustration of a movie of the** **Fig. 2C** **2 in group VII scanned at 15 minutes after the gel-filling.** A small amount of gel is filled into the circular incision performed on a segmental SSV in the left lateral thigh before scanning. The interrupted paramagnetic tracer by the incision (showed in Fig. 2C1) in the upstream, has passed through the incision and enhanced the entire downstream perivenous pathway by the help of gel.
(MOV)

Video S3 **Illustration of a movie of the** **Fig. 2C** **3 scanned at 35 minutes after the gel-filling.** The Gd-DTPA has clearly enhanced the filled gel (looked similar with Fig. 2F2) while the vessel wall is not broken and the intravenous blood has not leaked out.
(MOV)

Video S4 **Illustration of the increased pericardial effusion of a subject in group XVII dynamically detected by echocardiography.** A few amount of extra pericardial fluid can be seen (pointed by the white arrow) in anterior wall of right ventricle detected around 48 minutes after the 4 mL natural saline into KI3 in contrast to none found at the beginning.
(MOV)

Video S5 **Illustration of the increased pericardial effusion of the same subject in Video S4 detected by echocardiography.** A few amount of extra pericardial fluid can be seen (pointed by the white arrow) in posterior wall of left ventricle detected around 57 minutes after the 4 mL natural saline into KI3 in contrast to none found at the beginning.
(MOV)
