# Supplementary figures and images for: Fluid Flow along Venous Adventitia in Rabbits: Is It a Potential Drainage System Complementary to Vascular Circulations?
Source: PLoS One. 2012 Jul 26;7(7):e41395. doi: 10.1371/journal.pone.0041395 (PMC3406065; doi:10.1371/journal.pone.0041395)

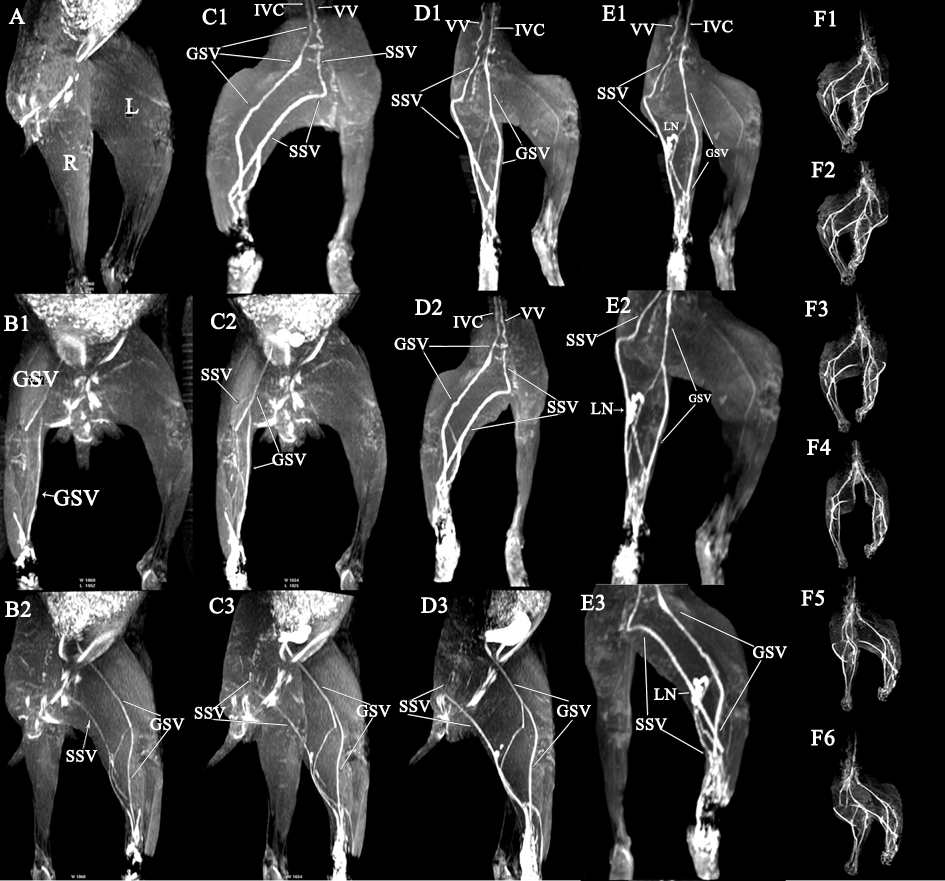

Supplement: Figure S1 — Illustrations of the pathways in lower limbs of rabbits by MRI. A, Blank view scans before injecting Gd-DTPA. B, Two angular views scan at 2 min after injection into right KI3 at 1–2 mm depth. GSV, SSV plus their main branches began to be displayed. C, Three angular views scan at 7 min after injection with increasing contrast’s signals. C1 is modified and shows right GSV, SSV and segments of inferior vena cava (IVC) and vertebral vein (VV). D, Scanned at 15 min, the signals in right GSV, SSV, IVC and VV are much more clearly. B2, C3 and D3 show tracer is collected increasingly in bladder. In the meanwhile, the signals in left GSV and SSV are very weak. E, Three angular views of the same subject are injected into right KI3 at 2–3 mm depth and scanned at 15 min from start in another day. Not only right GSV, SSV, IVC and VV but also popliteal lymph node (LN) and segments of afferent and efferent lymphatic collecting vessels (LV) are displayed together. F, Different views of angiography in lower limbs of the same subject scan in the other day, which coincide greatly with the pathways coming from KI3. (TIF) [file pone.0041395.s001.tif]

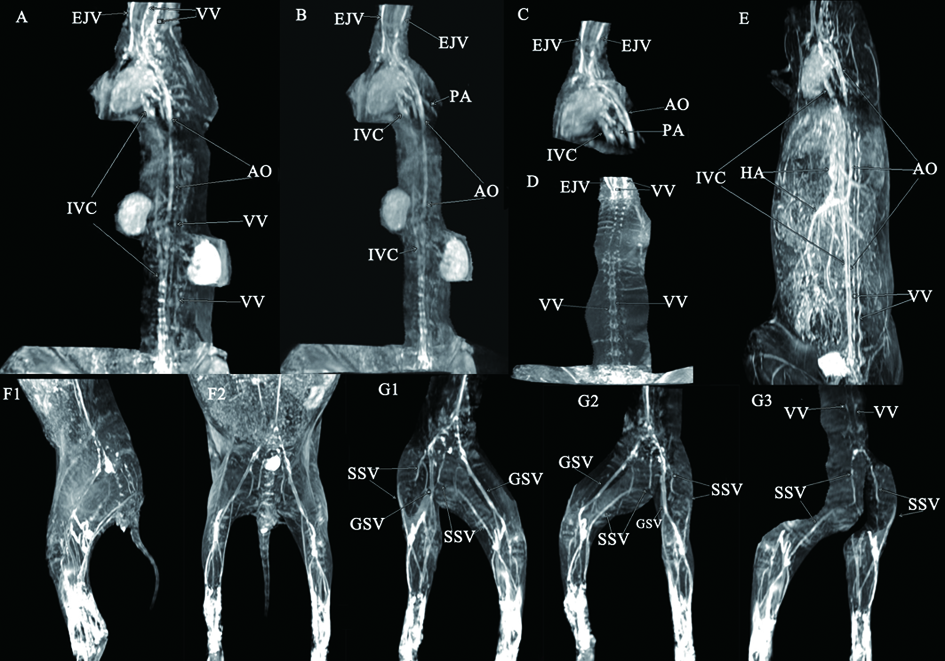

Supplement: Figure S2 — Illustrations of the pathways in intra-abdominal and intra-thoracic cavities and lower limbs of a rabbit scanned at 7 min after injecting tracer into right and left KI3 simultaneously. A, B, C, D show clearly IVC, external jugular veins (EJV), VV, pulmonary arteries (PA) and aorta (AO) in different views. F1, F2 show unabridged views of right and left GSV, SSV plus their main branches. G1, G2, G3 show different views of GSV, SSV and VV. E, Angiography by intravenous injection via auricular vein. IVC, VV, AO and hepatic arteries (HA) are clearly displayed. By comparing with the images of angiography by MRA, the pathways coming from KI3 coincide with the veins and arteries in trunk and lower limbs. (TIF) [file pone.0041395.s002.tif]

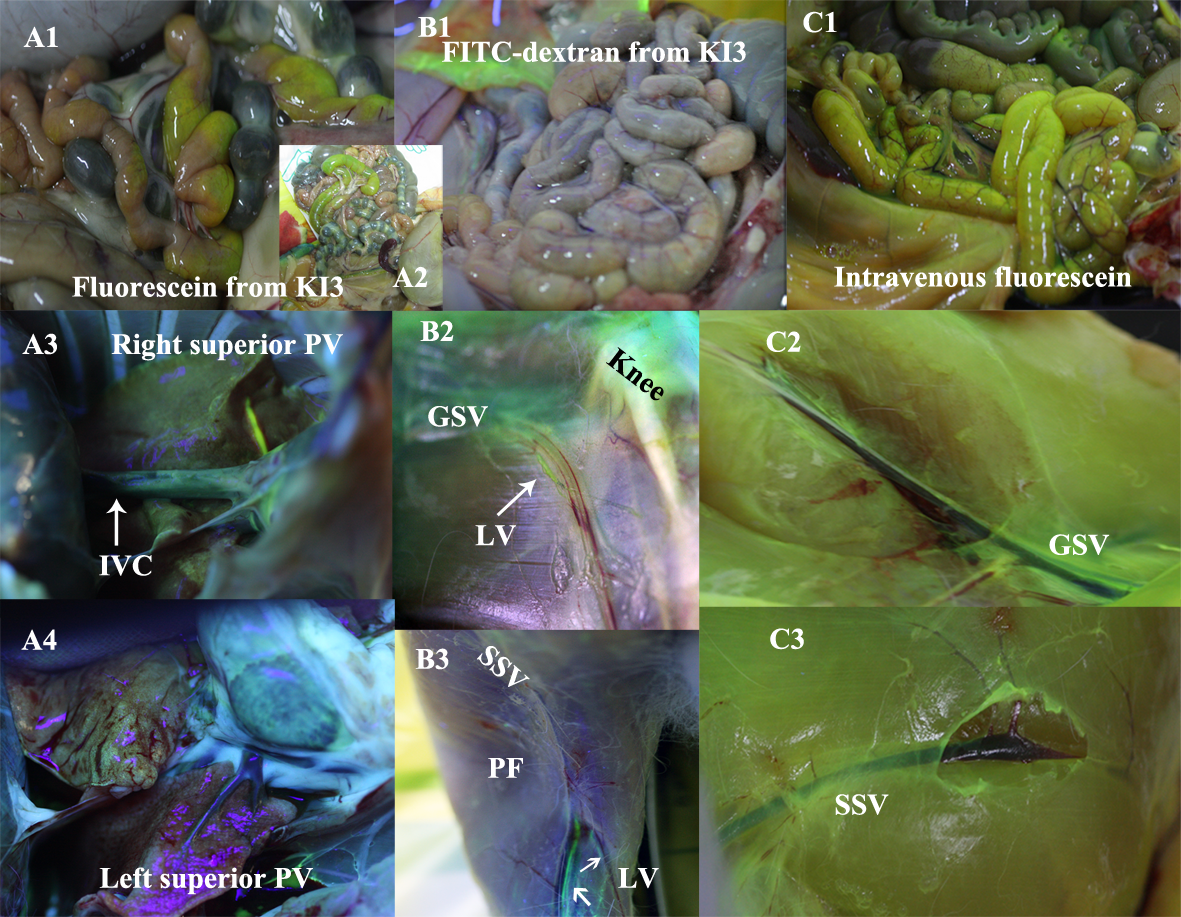

Supplement: Figure S3 — Illustrations of the differences among different tracers in intra-abdominal and intra-thoracic cavities. A1 shows only partial walls of small intestines are stained by a small amount of fluorescein sodium coming from KI3, and a longer intestine tube is stained by higher amount of fluorescein from KI3 showed in A2, in contrast to the walls of entire length of small intestines are stained by intravenous fluorescein sodium in C1, and no fluorescent signals found on small intestines in B1 by injecting FITC-dextran into KI3 in group IX. A3 show fluorescently stained right pulmonary veins in contrast to non-stained left pulmonary veins in A4 by fluorescein sodium from KI3 in group XVI. Lymphatic vessels (LV) are displayed in the vicinity of GSV in B2, SSVs in B3 (pointed by two white arrows) by FITC-dextran from KI3. B3 showed there were no collecting lymphatic vessels beside SSV above the level of popliteal fossa (PF). Note: B2, B3 also show there are no any venous walls stained by subcutaneous FITC-dextran injection into KI3. No increasingly accumulated strong fluorescent signals are found in the surrounding loose connective tissues along the entire length of GSV (C2) or SSV (C3) by intravenous fluorescein sodium into auricular vein in group XI. (TIF) [file pone.0041395.s003.tif]

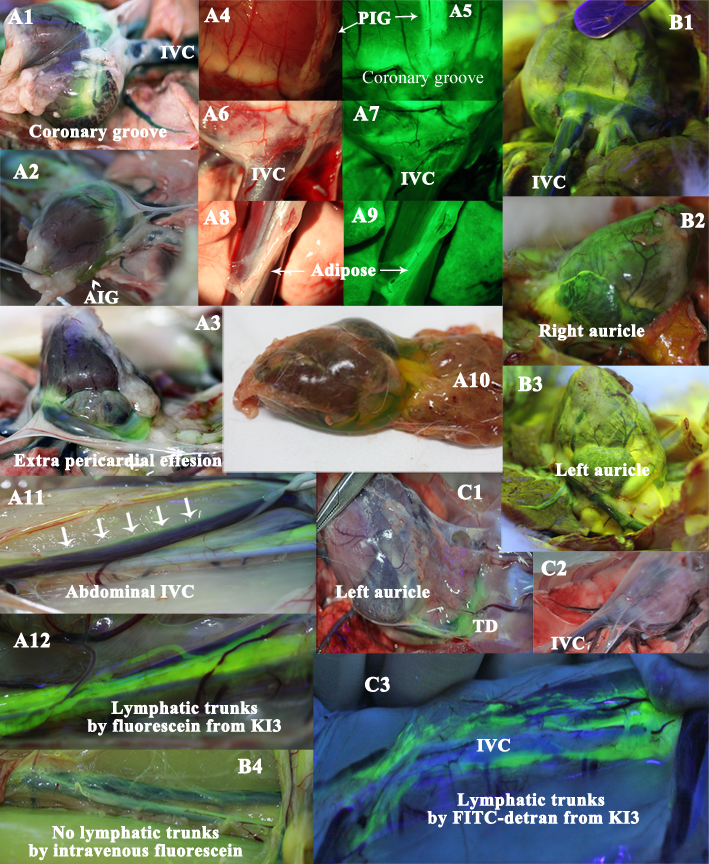

Supplement: Figure S4 — Illustrations of the differences among stained tissues over the heart and beside IVC by different tracers. Three views of the heart show the fluorescein sodium from KI3 have strongly accumulated and stained some tissues over the heart, including coronary groove in A1, a few amount of pericardial fluid in anterior inter-ventricular groove (AIG) and the superficial tissues on the left auricle in A2, and right auricle in A3 in a subject of group XVI. However, the other parts of the heart seem stained by less fluorescent signals in contrast to the strongly stained tissues all over the heart by intravenous fluorescein sodium in B1, B2 and B3 of group XI. In addition, the surfaces of IVC (A6, A7) including adipose tissues on it (A8, A9) and the tissues within coronary groove and posterior inter-ventricular groove (PIG) (A4, A5) are strongly stained by fluorescein sodium from KI3, which seems fluorescein sodium from KI3 has been transported along IVC and into three grooves to have stained pericardial fluid, speculatively. A10 shows a larger amount of pericardial fluid in the heart of a subject in group XVII with 5 mL injection into KI3, sacrificed at 90 minutes after the injection and the detection by echocardiography. Few amount of fluorescein sodium originating from KI3 are found over the loose connective tissues on IVC, pointed at by white arrows in A5. Only thoracic duct passing through the cardiac base is visualized by FITC-dextran from KI3, showed in C1, and no fluorescent signals on IVC in C2 of group IX. The lymphatic trunks beside IVC are displayed by either fluorescein sodium from KI3 (A12) or FITC-dextran from KI3 (C3), but not by intravenous fluorescein sodium (B4). (TIF) [file pone.0041395.s004.tif]
